# Ecological responses of soil bacteria to rapid and slow temperature fluctuations

**DOI:** 10.1098/rspb.2026.1267

**Published:** 2026-10-07

**Authors:** Xin-Feng Zhao, Yu-Chen Qiu, Lin Li, Chang Yang, Xu Zheng, Wen-Sheng Shu, Yi-Qi Hao

**Affiliations:** Guangzhou Key Laboratory of Subtropical Biodiversity and Biomonitoring, Guangdong Provincial Key Laboratory of Biotechnology for Plant Development, School of Life Sciences, South China Normal University, Guangzhou 510631, People's Republic of China

**Keywords:** fluctuating temperature, community ecology, temperature transition, thermal response

## Abstract

Fluctuating temperatures profoundly affect microorganisms’ coexistence, biogeographic distributions and responses to future climate change. However, how microorganisms respond to fluctuating temperatures with different frequencies in natural communities remains unknown. We conducted a 120-day temperature manipulation experiment with soil-bacterial communities subjected to constant low, medium and high temperatures, as well as fluctuating temperatures with three different frequencies. Four groups of temperature-responsive species were identified, including species favoured by constant high or low temperature, and species favoured or disfavoured by rapid temperature fluctuations. Further analyses confirmed that species in the first two groups only responded to the constant temperatures, and their performances at all fluctuating temperatures were roughly equal to their time-averaged performances at high and low temperatures. Species in the last two groups showed specific responses to frequent temperature transitions. Our results revealed that the performances of bacterial species under slow temperature fluctuation closely resembled their time-averaged performances at high and low temperatures, but deviated from those at medium temperature, due to the nonlinearity of temperature-dependent performances. Our study highlights that soil bacteria show unique responses to both rapid and slow temperature fluctuations compared to medium temperature, which is crucial to understand microbial community dynamics in variable thermal environments.

## 1. Introduction

Temperature is an environmental factor of great biological significance in determining the performances of microorganisms [1–5]. One of the important attributes of the thermal environment in nature is that temperature continuously fluctuates at different time scales. Microorganisms have short generation times, which enable them to sense and rapidly respond to temperature changes [6,7]. Therefore, the diurnal, within-season, seasonal and inter-annual fluctuations in temperature should all have pronounced influences on microbial performances and community dynamics. Considering that microorganisms comprise a large portion of biomass on earth [8,9], are largely responsible for biogeochemical processes and play a key role in climate feedback [10,11], it is crucial to understand how microorganisms respond to fluctuating temperatures in natural communities. In practice, most empirical studies of microbial biogeography only focus on mean temperature as a proxy for the thermal environment to investigate the variations in community composition along temperature gradients [12–14]. There have also been extensive experiments investigating the effects of increased temperature variability on microbial community composition and ecosystem functioning in the context of global warming [15–17]. However, how microorganisms in natural communities respond to fluctuating temperatures with different frequencies from the mean temperature remains poorly understood.

Fluctuating temperatures are composed of not only periods of different temperatures but also the transitions between them. Studies focusing on the amplitude of temperature fluctuation primarily reveal the effects of component temperatures, especially the maximum and minimum temperatures, on microbial performances and community dynamics [15–17]. By contrast, temperature transition (the temperature shifts between maximum and minimum temperatures during temperature fluctuations) is a potentially important and unique component of fluctuating temperatures, but its effects have rarely been studied in natural microbial communities. Nonetheless, studies of experimental evolution have suggested that species at fluctuating temperatures might specifically adapt to the temperature transition [18–21]. For example, the growth advantages of bacterial lineages evolved at fluctuating temperatures are only apparent when measured at fluctuating temperatures, and the performances measured across constant temperatures incorrectly predict their performances at fluctuating temperatures [19,20,22]. There is also evidence to the contrary suggesting that microbes cope with fluctuating temperatures via adapting to the component temperatures. For example, a long-term selection experiment has found that the evolved lines of *Escherichia coli* experiencing alternating temperatures between 32°C and 42°C have significantly improved fitness at each of the component temperatures, but show no significant improvement to the sudden temperature transitions [23]. These findings suggest that microorganisms adopt various strategies to cope with fluctuating temperatures. However, how microorganisms respond to the component temperatures and temperature transitions under temperature fluctuations in natural communities remains largely unexplored.

By synthesizing theories and experimental evidence, we propose two processes that can result in discrepancies among microbial species in response to rapid/slow temperature fluctuations and mean temperature in microbial communities. Firstly, there are microbial species that are either specifically adapted to or sensitive to temperature transitions [19–22]; therefore, temperature transitions might induce specific responses of microorganisms. We anticipate this process would be more significant when microorganisms experience more frequent temperature transitions under rapid temperature fluctuations.

Secondly, the assumed equivalence between fluctuating and mean temperatures in determining the performances of microorganisms is based on a prerequisite that the relationships between species’ performances and temperatures are linear [24–26], which is not supported by experimental evidence [4,27,28]. When the frequency of temperature fluctuation is low, microorganisms experience alternate long-term high and low temperature phases with minimized effects of temperature transitions. The performances of microorganisms are expected to be equivalent to their time-averaged performances at constant temperatures [29]. Nevertheless, the nonlinearity of thermal performance curves will inevitably result in significant deviations between time-averaged performances and performances at mean temperature, which is an intrinsic mathematical property of nonlinear averaging known as Jensen's inequality [24,25,30]. So far, a large number of studies on microbial reaction norms have found that the relationships between performances (e.g. growth rates, enzymatic activities, metabolic rates) of microorganisms and temperatures are nonlinear [4,27,31,32]. Therefore, we expect that even under slow temperature fluctuations, microorganisms will exhibit different responses compared to those under a constant mean temperature.

Topsoil-bacterial communities are ubiquitous and play essential roles in nutrient cycling across divergent terrestrial ecosystems [8,33]. They experience diurnal, within-season, seasonal and inter-annual fluctuations in temperature. Especially, the responses of soil-bacterial species to temperature are critical for projections of the feedbacks between carbon cycle and climate change [34,35]. Therefore, it is of great significance to examine the responses of soil-bacterial species to rapid and slow temperature fluctuations. Here, we conducted a 120-day temperature manipulation microcosm experiment with topsoil. The temperate forest topsoil collected from northern China was subjected to constant high (H, 26°C), low (L, 16°C), medium (M, 21°C) temperatures or fluctuating temperatures switching between 26 and 16°C with high (HF), intermediate (IF) or low (LF) frequencies (switching every half day, every 5 days, or every 20 days, respectively) for 120 days. The composition of soil-bacterial communities during incubation was assayed using a high-throughput amplicon sequencing method. The goal of our study is to identify temperature-responsive species and elucidate the ecological processes that drive their distinct responses to different temperature regimes.

## 2. Methods

### (a) Soil collection and preparation

The soil was collected from a typical temperate deciduous forest in northern China (42°31′36.5″ N, 125°43′12.7″ E, H 518 m), with an average annual temperature of 5.1°C, annual precipitation of 632 mm and significant diurnal, within-season and seasonal temperature fluctuations (supplementary material, table S1). Soil was sampled in June 2020, from the top 15 cm of a 1 m × 1 m square area. The 10 kg of soil was then transferred to disposable sterile bags and immediately transported to the laboratory in coolers. The soil was sieved with 5-mm filters and carefully homogenized prior to the temperature manipulation experiment.

### (b) Soil microcosm experiment

We established three constant temperature treatments and three fluctuating temperature treatments with different fluctuation frequencies in our experiment. For the constant temperature treatments, we selected 16°C as the low incubation temperature (L), 21°C as the medium incubation temperature (M) and 26°C as the high incubation temperature (H). In the fluctuating temperature treatments, temperatures switched between 26°C and 16°C at high frequency (HF, switching every half day), intermediate frequency (IF, switching every 5 days) and low frequency (LF, switching every 20 days), respectively. The entire experiment spanned 120 days. The temperature settings represented the actual temperature variations experienced by soil bacteria during the growing season (supplementary material, table S1). To ensure effective comparisons between treatments, the temperatures and accumulated time of high- and low-temperature phases were consistent across all three fluctuating temperature treatments.

There were eight replicates in each treatment, yielding 48 soil microcosms in total. Each soil microcosm contained 100 g of soil in a 250 ml sterilized glass bottle. These soil microcosms were subjected to the corresponding temperature treatments described above. Sterilized water was added weekly to each microcosm to maintain the original soil water content (28.6%). Bottle caps of all microcosms remained loose throughout the experiment, and microcosms were aerated for 15 min on a clean bench every 2 d to maintain aerobic conditions and prevent oxygen depletion. Soil samples were collected at the beginning and every 40 days during the experiment (day 0: T0; day 40: T1; day 80: T2; day 120: T3). Two grams of soil was collected from each microcosm at each time point, sealed in 15-ml sterilized centrifuge tubes and stored at −80°C.

### (c) 16S rRNA gene sequencing

The soil-bacterial community composition was analysed through a high-throughput amplicon sequencing method targeting the V4 region of the 16S rRNA genes, as previously described [29] (supplementary material, method S1). The raw sequence data were deposited at the National Center for Biotechnology Information (NCBI) Sequence Read Archive SRA under BioProject PRJNA 1233643. The raw data were processed using QIIME2 2019-07 [36], following a standard data processing and denoising pipeline (supplementary material, method S1). The Deblur algorithm was employed to identify amplicon sequence variants (ASVs), which were defined as ‘species’ in our study [37,38]. Bacterial species whose relative abundance across all samples was less than 0.001% were excluded to minimize the impact of sequencing errors, resulting in a total of 5930 retained species.

### (d) Statistical analysis

The data analyses were all conducted on compositional data post-rarefaction (27 420 sequences per sample), thus differences in the number of reads of each species across treatments or time points represented differences in relative abundance. Neither temperature treatment nor time had a significant effect on the density of bacteria in soil (supplementary material, method S2, figure S1, table S2); therefore, changes in relative abundance could adequately represent the performances of soil bacteria in our study, and the number of reads of each species was referred to as the abundance here. All of the statistical analyses were conducted in R v. 4.0.1 [39].

To quantify dissimilarities in species composition among soil-bacterial communities, Bray–Curtis distances were computed. The permutational multivariate analysis of variance (PERMANOVA) was employed to assess the influences of time, treatment and their interaction on community composition (permutations = 9999) using the ‘vegan’ package in R (v. 2.5-6). One outlier community in the HF treatment at T0 (supplementary material, figure S2A) was excluded from all analyses. Species richness at the end of the experiment was compared among the six treatments using Tukey's multiple comparison test.

Temperature-responsive species were identified by comparing the abundance of common species between different temperature treatments after 120 days of incubation. Considering that the differences in abundance of rare species were very sensitive to sampling effects, only common species (relative abundance across all samples >0.01%) were included in the analyses, which collectively accounted for 84.8% of the total abundance. Differences in abundance were measured as log2-fold changes (LFCs) using the algorithm DESeq2 (‘DESeq2’ package, version 1.28.1) with a negative binomial frequency distribution model, and the *p*-values were adjusted for multiple tests following Benjamini and Hochberg's procedure [40,41]. The specific procedures of identifying temperature-responsive species were as follows (supplementary material, figure S3). Firstly, we compared the abundance of each common species between the H and L treatments at the end of the experiment, and identified H- and L-superior species as those that had significantly higher abundance at high or low temperature, respectively. Secondly, we compared the abundance of the remaining common species in each of the three fluctuating temperature treatments with that in the H and L treatments, and identified species responding to fluctuating temperatures as those that had significant differences in abundance between any fluctuating temperature treatment (LF, IF or HF) and any constant temperature treatment (H or L). For instance, species with significantly higher abundance in the LF treatment than in the H and/or L treatments were defined as LF-superior species (IF-superior species for the IF treatment, and HF-superior species for the HF treatment); meanwhile, species with significantly lower abundance in the LF treatment than in the H and/or L treatments were defined as LF-inferior species (IF-inferior species for the IF treatment, and HF-inferior species for the HF treatment). Due to similarity in community composition (figure 1A), the HF and IF treatments were hereafter referred to as the rapid fluctuation (RF) treatment. Accordingly, the IF- and HF-superior species were collectively referred to as RF-superior species, and IF- and HF-inferior species as RF-inferior species for further analyses (supplementary material, dataset S1).

**Figure 1.**
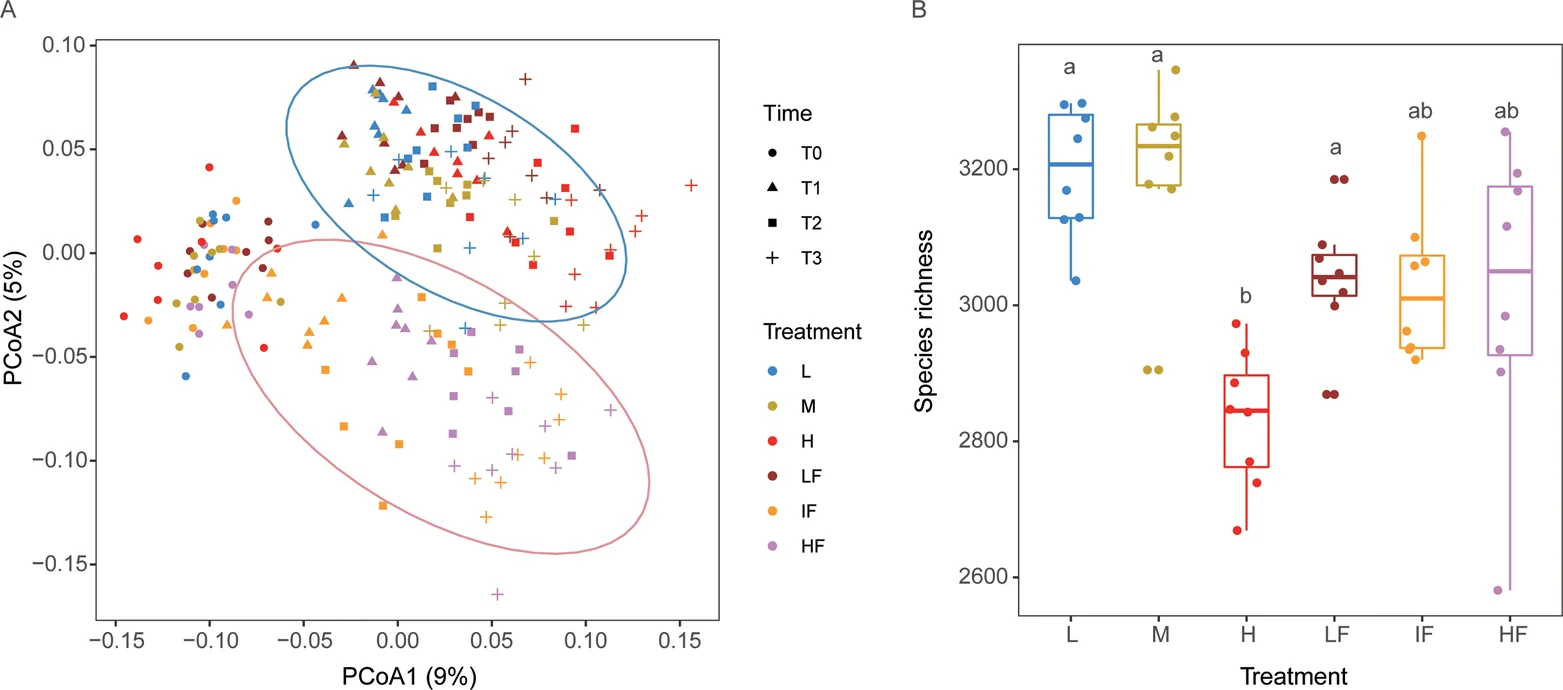
Community composition and diversity differences across different temperature treatments. (A) Metric multidimensional scaling ordination of soil-bacterial communities by principal coordinate analysis (PCoA). Each point represents an experimental soil-bacterial community (*n* = 191; one outlier community in the HF treatment at T0 was discarded in this analysis; supplementary material, figure S2A). Distances among communities represent Bray–Curtis dissimilarities. The communities are coloured based on the treatments, and communities at different time points are indicated by shapes. The soil-bacterial communities diverged into two distinct groups in our experiment (supplementary material, figure S5); one group consisted of three constant temperature treatments and the LF treatment (95% normal confidence ellipses, blue circle), while the other group consisted of the IF and HF treatments (95% normal confidence ellipses, pink circle). (B) Species richness of soil-bacterial communities at the end of the experiment. Scatter points within each treatment group represent raw values from individual replicates. The lower and upper ends of the boxes represent 25% and 75% of the range, respectively; the central line indicates the median; and whiskers represent ±1.5 × interquartile range (IQR, defined as the upper quartile minus the lower quartile). Different lowercase letters denote significant differences in species richness between six treatments (Tukey's multiple comparison, *p*_adj_ < 0.05; supplementary material, table S6).

We then investigated the phylogenetic conservatism of soil-bacterial responses to different temperature regimes. The phylogenetic signal of each temperature-responsive trait was tested using Pagel's *λ* test [42]. The degree of phylogenetic clustering of species belonging to each temperature-responsive group was examined using the net relatedness index (NRI) and nearest taxon index (NTI) [43], and the significance of phylogenetic clustering was tested by comparing the observed indices to those of null models by shuffling tip labels (9999 random permutations).

Log2-fold changes between the end and beginning of the experiment were calculated for each temperature-responsive species to represent its performance in each treatment. For predicting the performances of temperature-responsive species at three fluctuating temperatures, we employed two predictors: (i) the time-averaged performances at fluctuating temperatures based on the performances at the high (LFC_H_) and low (LFC_L_) temperatures, that is, ½ × LFC_H_ + ½ × LFC_L_; and (ii) the performance at the medium temperature (LFC_M_). The relationships between the observed LFCs and the predicted LFCs in the fluctuating temperature treatments were analysed with linear regression. To assess differences between the observed LFCs and predicted LFCs in the fluctuating temperature treatments, mixed-effects linear models were conducted with the category of LFCs (predicted or observed) as a fixed factor and species identity as a random factor for H-superior, L-superior, RF-superior and RF-inferior species, respectively.

Subsequently, we analysed the nonlinear dependence of species’ performance on temperature, and we focused on the LF treatment with minimized effects of temperature transitions. Firstly, we assessed the differences in abundance (measured as LFCs) between the end and the beginning of the experiment for each common species in the L, M, H and LF treatments. Species exhibiting significant increases in abundance in one or more treatments without significant decreases in abundance in any treatment were categorized as ‘species with increased abundance’. Species exhibiting significant decreases in abundance in one or more treatments without significant increases in abundance in any treatment were classified as ‘species with decreased abundance’. Only one species showed inconsistent significant changes in abundance among treatments, which was not included in the analyses (supplementary material, dataset S2). Then, the nonlinear relationships between species performances and temperatures were analysed separately for species with increased or decreased abundance. Specifically, the differences in LFCs between the L, M, H, LF treatments and time-averaged LFCs were tested using mixed-effects linear models, with temperature treatment as a fixed factor and species identity as a random factor.

Next, we analysed the performances of different groups of temperature-responsive species at constant and fluctuating temperatures. Two-tailed one-sample *t*-tests were used to determine whether the performances (LFCs over time) of H-superior, L-superior, RF-superior and RF-inferior species in each treatment were significantly different from zero; *p*-values were adjusted following the Benjamini–Hochberg procedure. Differences in performances (LFCs over time) among the four groups of temperature-responsive species in each treatment were tested using ANOVA, and multiple comparisons were then conducted by Tukey's HSD tests.

## 3. Results

### (a) Community divergence under different temperature regimes

Temperature treatments induced significant changes in soil-bacterial community composition within 40 d (figure 1A; supplementary material, figure S2B, table S3), and the community divergence was further enlarged during the next 80 d (figure 1A; supplementary material, table S4). In general, soil-bacterial communities diverged into two main groups. Specifically, communities at fluctuating temperatures with high and intermediate frequencies clustered separately from those at constant temperatures and fluctuating temperatures with low frequency (figure 1A; supplementary material, figures S2 and S5). At the end of the experiment, pairwise comparisons showed that communities under different temperature regimes were all significantly different from each other (supplementary material, table S5), except those from the two rapid temperature fluctuation treatments (HF and IF, collectively referred to as rapid temperature fluctuation treatments, RF), which shared similar community composition (supplementary material, table S5).

Temperature treatments also induced significant effects on species richness of bacterial communities. Species richness in the L and M treatments showed no significant change over time, whereas it declined most markedly in the H treatment. The three fluctuating temperature treatments exhibited weaker declining trends compared to the H treatment (supplementary material, figure S4). Accordingly, by the end of the experiment, species richness was significantly lower in the H treatment compared with the L and M treatments, while richness under the three fluctuating temperature regimes fell to intermediate levels (figure 1B). Differences in species richness declining trends across the H, L and LF treatments were consistent with a previous long-term temperature fluctuation experiment of soil-bacterial communities [29].

### (b) Classification of temperature-responsive species into four groups

To reveal the responses of soil bacteria to different temperature regimes, we targeted species which had significant differences in abundance between temperature treatments at the end of the experiment (measured as LFCs). We were particularly interested in species favoured by constant high or low temperature, and species favoured or disfavoured by fluctuating temperatures (supplementary material, figure S3). In total, 45 H- and 49 L-superior species were identified, which accounted for 4.20% and 2.88% of the total abundance (figure 2A; supplementary material, dataset S1). In addition, a total of 103 (46 + 15 + 24 + 18) species responded to the IF treatment, and 92 (15 + 29 + 18 + 30) species responded to the HF treatment, with 33 (15 + 18) overlapped species (figure 2A; supplementary material, dataset S1). The IF- and HF-superior species were collectively referred to as RF-superior species, and IF- and HF-inferior species as RF-inferior species for further analyses. This merging was based on the following two facts: (i) the abundance of 159 out of these 162 species in the IF treatment was not significantly different from that in the HF treatment (supplementary material, dataset S1), (ii) the composition of communities in the IF treatment was not significantly different from that in the HF treatment at the end of experiment (supplementary material, figure S2D, table S5). The 90 RF-superior species and 72 RF-inferior species which were favoured or disfavoured by rapid temperature fluctuations accounted for 9.46% and 5.29% of the total abundance, respectively (figure 2A; supplementary material, dataset S1). By contrast, only two species were identified as LF-inferior species and one as LF-superior species (figure 2A; supplementary material, dataset S1); thus, no further statistical analyses were conducted on them.

**Figure 2.**
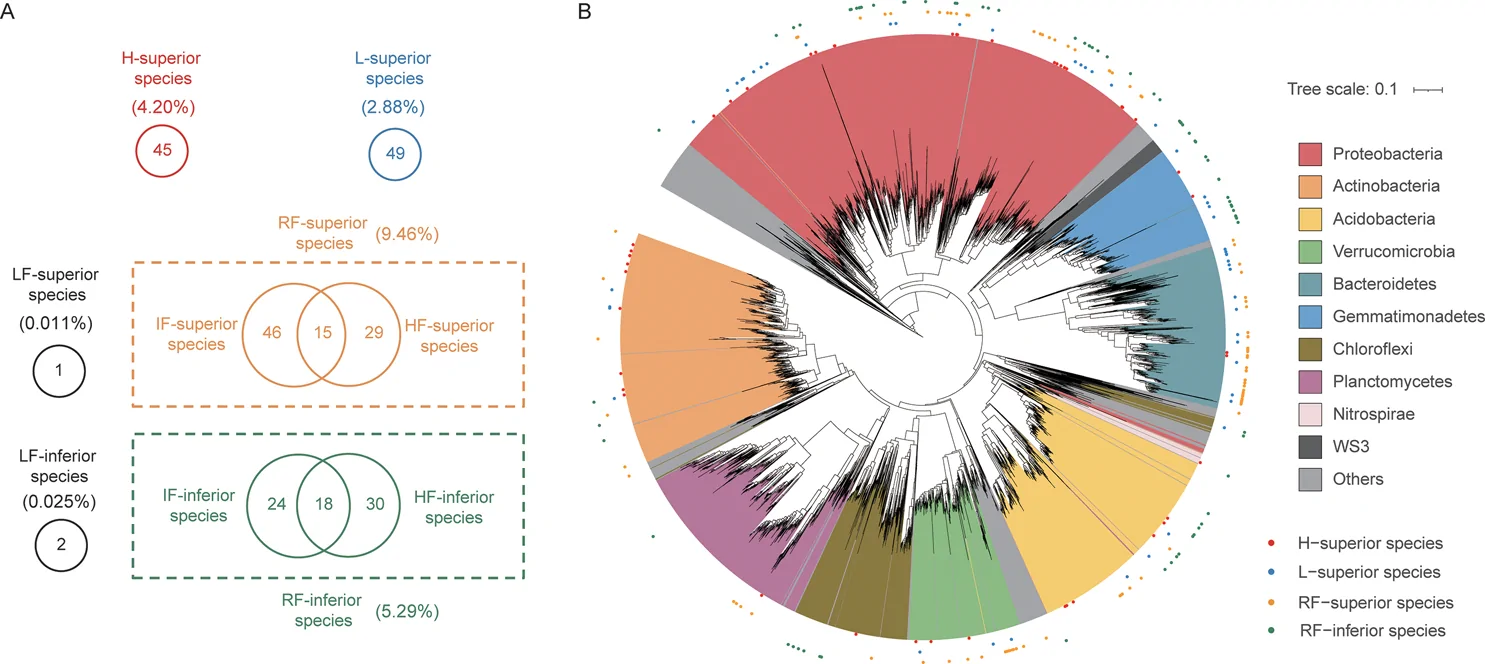
Classification of soil-bacterial species into four different temperature-responsive groups. (A) Classification of temperature-responsive species based on the differences in their abundance between different temperature treatments at the end of the experiment (supplementary material, figure S3). The number of species and corresponding collective relative abundance of species in each temperature-responsive group are provided. The IF- and HF-superior (-inferior) species are collectively referred to as RF-superior (-inferior) species. (B) Phylogenetic distribution of four groups of temperature-responsive species on the local species pool tree containing 5930 species. Phyla are indicated by branch colours. The groups of temperature-responsive species are indicated by the colours of points around the phylogenetic tree. The taxonomic assignment does not match tree topology for a small portion of species, and no manual corrections are made.

### (c) Phylogenetic conservatism of responses of bacterial species to different temperature regimes

The major soil-bacterial phyla in our study, including Acidobacteria, Actinobacteria, Bacteroidetes, Proteobacteria, and Verrucomicrobia, all contained H-superior, L-superior and RF-superior species (figure 2B; supplementary material, dataset S1). The phylogenetic signal of H-superior species was non-significant (supplementary material, table S7; Pagel's tests, *λ* = 0.0066, *p* = 0.21). The phylogenetic signals of L-superior, RF-superior and RF-inferior species were significant but very weak, suggested by the low *λ* values (supplementary material, table S7; Pagel's tests, all *λ* < 0.05, *p* < 0.05). The phylogenetic relatedness tests showed that species in all four temperature-responsive groups clustered near the tips of the phylogenetic tree (supplementary material, table S8; all NTI > 2, *p* < 0.01). In addition, H-superior and RF-inferior species also clustered at deeper phylogenetic branches (supplementary material, table S8; all NRI > 2, *p* < 0.01), but the distributions of L-superior species (supplementary material, table S8; NRI = −0.18, *p* = 0.59) and RF-superior species (supplementary material, table S8; NRI = −0.55, *p* = 0.71) across the whole phylogenetic tree were indistinguishable from the random distributions. Overall, these results indicated that the responses of soil bacteria to temperatures were phylogenetically conserved, but at a shallow level.

Furthermore, we applied the widely used *r*- (copiotrophic species) and *K*-selected (oligotrophic species) categories [44–47] to investigate the potential associations between life-history strategies and responses to different temperature regimes. We found that RF-superior species were more likely to be copiotrophs (supplementary material, table S9; copiotroph:oligotroph ratio = 2.36, chi-square test, *χ*^2^ = 13.76, *df* = 1, *p* < 0.001).

### (d) Specific responses of RF-superior and RF-inferior species to temperature transitions

We examined the relationships between the performances of temperature-responsive species (changes in abundance of each species during the whole experiment, measured as LFCs over time) at fluctuating temperatures and those at constant temperatures. The time-averaged performances of temperature-responsive species, based on their performances at high and low temperatures, were used to predict their performances at fluctuating temperatures. We found that for all temperature-responsive species, their performances under slow temperature fluctuation could be well predicted by their performances at high and low temperatures (figure 3A,D). There were strong linear relationships between their observed and predicted performances in the LF treatment, with slope nearly equal to one and intercept close to zero (figure 3A,D). Furthermore, there was no significant deviation between their observed and predicted performances in the LF treatment (supplementary material, table S10). In the IF and HF treatments, there were still no significant differences between observed and predicted performances for H- and L-superior species (supplementary material, table S10), and the linear relationships between their observed and predicted performances were also significant (figure 3B,C). However, the time-averaged performance was no longer a reliable predictor of the observed performance under rapid temperature fluctuations for both RF-superior and RF-inferior species. RF-superior species exhibited significantly better performances, while RF-inferior species showed significantly worse performances than the predicted performances in the IF and HF treatments (figure 3E,F; supplementary material, table S10).

**Figure 3.**
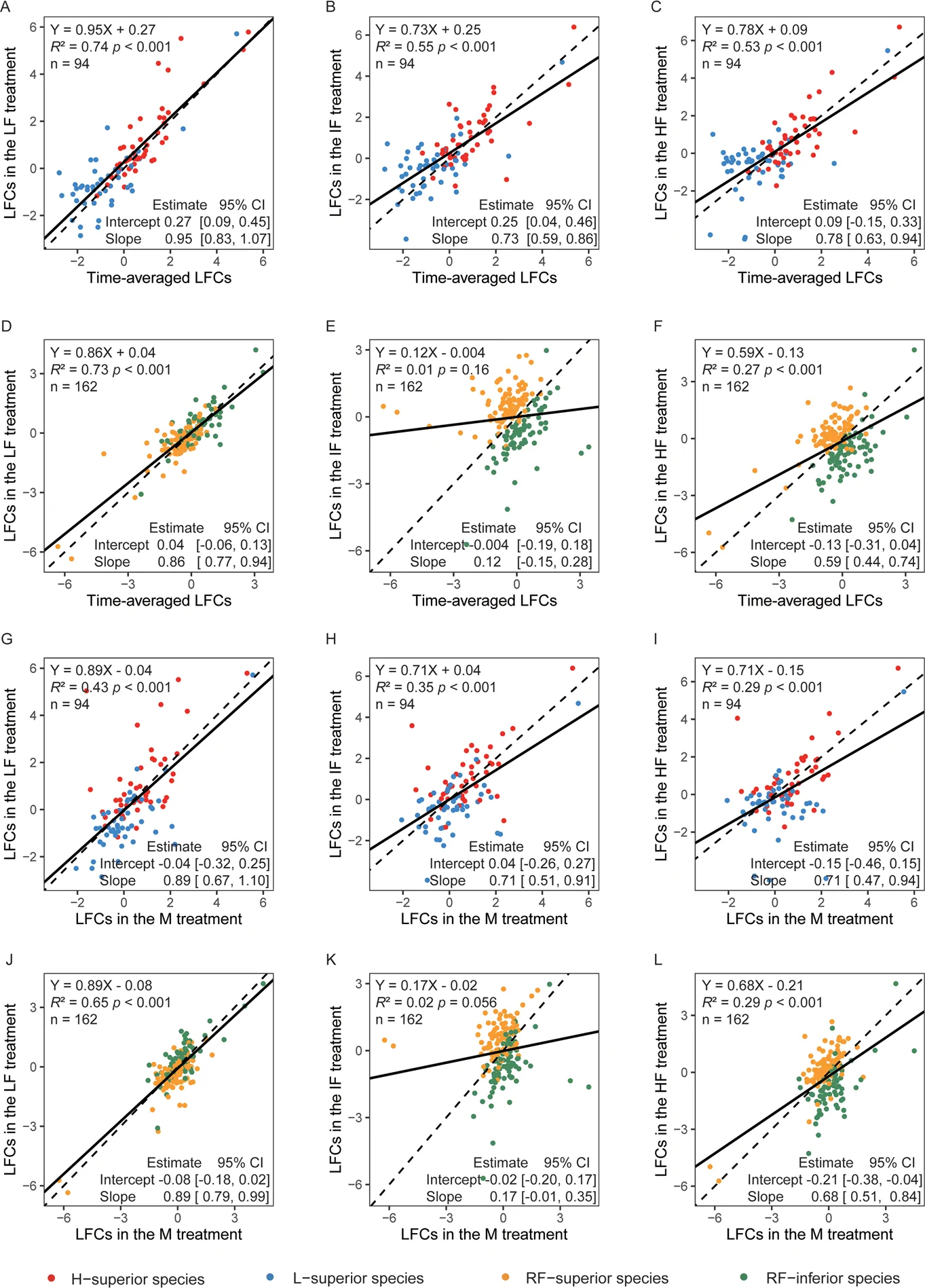
Relationships between the observed performances (log2-fold changes across the whole experiment, LFCs) of temperature-responsive species in the three fluctuating temperature treatments (LF, IF, HF) and two predictors ((A–F) the time-averaged performances and (G–L) the observed performances in the M treatment). The time-averaged performances are calculated based on species’ performances across the whole experiment in the H and L treatments, i.e. the time-averaged LFC = ½ × LFC_H_ + ½ × LFC_L_. The dashed lines indicate the situations where the observed LFCs are exactly equal to the predicted LFCs, and the solid lines indicate the linear relationships between the observed and predicted LFCs. Statistics of linear relationships between the observed and predicted LFCs are provided at the upper left corner of each panel (supplementary material, table S11). The 95% confidence intervals of intercepts and slopes are provided at the lower right corner of each panel. The groups of temperature-responsive species are indicated by the point colours.

We also examined the relationships between the performances of temperature-responsive species in the M treatment and those at fluctuating temperatures. Similarly, the performances of RF-superior and RF-inferior species in the IF and HF treatments showed the greatest deviations from those in the M treatment (figure 3K,L; supplementary material, table S10). However, significant deviations were also observed between the performances in the M treatment and those in the LF treatment for H-superior, L-superior and RF-superior species (figure 3G,J; supplementary material, table S10). Additionally, compared to the time-averaged performances, the performances in the M treatment explained a relatively smaller proportion of variation in performances for RF-superior and RF-inferior species in the LF treatment and for H- and L-superior species in all fluctuating temperature treatments (figure 3; supplementary material, table S11). In summary, the performances at medium temperature had weaker predictive power on performances at fluctuating temperatures compared to time-averaged performances.

### (e) The nonlinearity of bacterial performances across temperature gradient

We hypothesized that the deviations between bacterial performances at medium temperature and the time-averaged performances resulted from the nonlinearity of bacterial performances across the temperature gradient. Specifically, we expected that the changes in abundance should show an accelerating increase as temperature increased (figure 4A,B), which led to three deductions as follows: (i) the changes in abundance at medium temperature should be more similar to those at low temperature than to those at high temperature (figure 4A,B); (ii) for species with an increase in abundance, the time-averaged changes in abundance in the LF treatment should be larger than those in the M treatment (figure 4A) and vice versa for species with a decrease in abundance (figure 4B); (iii) the observed changes in abundance in the LF treatment should be more similar to the time-averaged changes than to those in the M treatment (figure 4A,B).

**Figure 4.**
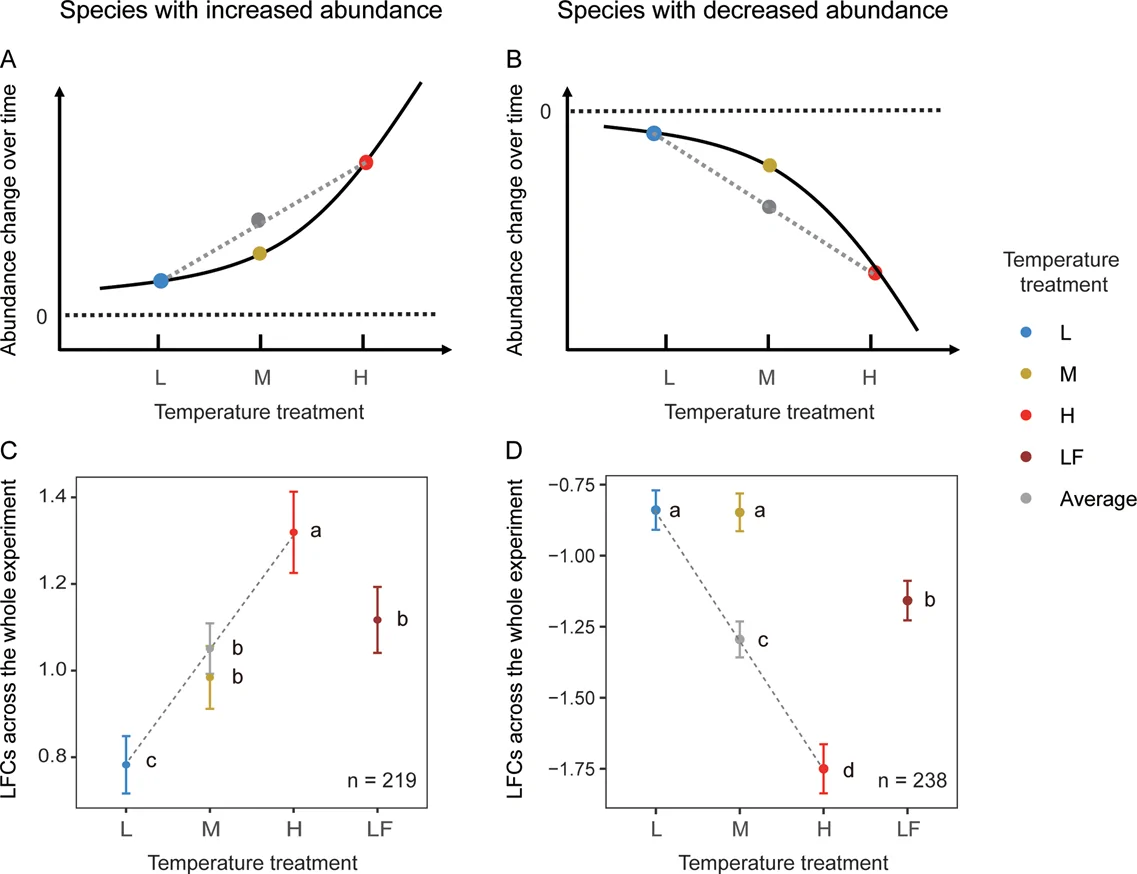
(A,B) A schematic illustration of the hypothetical non-linear dependence of soil-bacterial performances on temperature and (C,D) the corresponding experimental evidence. The dashed grey lines indicate the linear relationships between species’ performances and temperature, where species’ performances at the medium temperature would be equal to the time-averaged performances of species at the low and high temperatures (grey points). For non-linear dependence of soil-bacterial performances on temperature (the solid black curves), species’ performances change more from medium to high temperature than from low to medium temperature, causing bacterial performances at the medium temperature to deviate from the time-averaged performances (A,B). The experimental evidence supports non-linear dependence of soil-bacterial performances on temperature (C,D). The LFCs (mean ± s.e.) across the whole experiment of each species are used to represent bacterial performances in different temperature treatments, and the time-averaged performances are calculated based on species’ performances across the whole experiment in the H and L treatments, i.e. the time-averaged LFC = ½ × LFC_H_ + ½ × LFC_L_. Point colours denote temperature treatments. Different letters denote significant differences in LFCs between treatments for species with increased or decreased abundance, respectively (mixed-effects linear model, *p* < 0.05; supplementary material, table S12).

The analyses of nonlinearity of bacterial performance were conducted for species with increased or decreased abundance, separately. A total of 219 species were categorized as species with increased abundance (figure 4C). Additionally, 238 species were classified as species with decreased abundance (figure 4D). As expected, we found that for species with decreased abundance, all three deductions were confirmed, and the results were significant. (i) Their LFCs in the M treatment were not significantly different from those in the L treatment, but significantly larger than those in the H treatment (figure 4D; supplementary material, table S12). (ii) Their time-averaged LFCs were significantly smaller than those in the M treatment (figure 4D; supplementary material, table S12). (iii) The differences between their LFCs in the LF treatment and time-averaged LFCs were significantly smaller than those between their LFCs in the LF treatment and LFCs in the M treatment (figure 4D; supplementary material, table S12). For species with increased abundance, although they showed the patterns as we expected, all three results were not significant (figure 4C; supplementary material, table S12).

### (f) Competitive hierarchy of four temperature-responsive groups under different temperature regimes

To investigate how temperature regimes alter the competitive hierarchy of four groups of temperature-responsive species, we compared the bacterial performances among the four temperature-responsive groups in each temperature treatment. We found that the competitive superiority of temperature-responsive species varied depending on the thermal environments (figure 5). Specifically, H-superior species had the greatest competitive advantages under nearly all temperature regimes except for the low temperature, exhibiting the largest increases in abundance over time (figure 5; supplementary material, tables S13 and S14). By contrast, L-superior species were the most inferior competitors, showing significant decreases in abundance at high and all fluctuating temperatures (figure 5; supplementary material, table S13). Besides, RF-superior species gained competitive advantages relative to RF-inferior species under rapid temperature fluctuations, whereas RF-inferior species outperformed RF-superior species in the H, L and LF treatments (figure 5; supplementary material, table S14).

**Figure 5.**
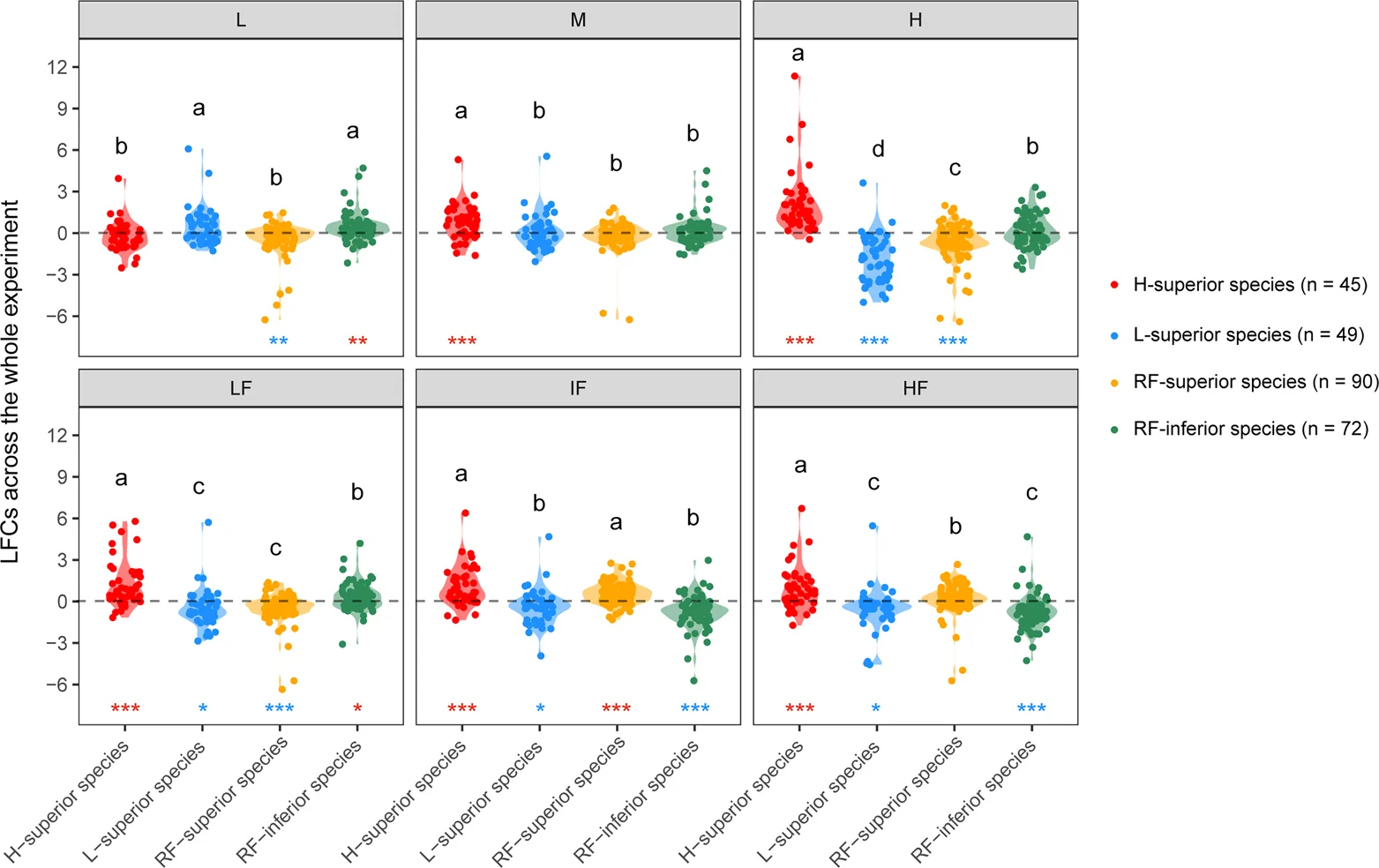
Performances (log2-fold changes across the whole experiment, LFCs) of temperature-responsive species in each temperature treatment. The asterisks at the bottom of the panels indicate significant differences between the values of LFCs and zero (two-tailed one-sample *t*-test; *p*-values are adjusted following the Benjamini–Hochberg procedure, *p_adj_ < 0.05, **p_adj_ < 0.01, ***p_adj_ < 0.001; red colour: significantly larger than zero, blue colour: significantly smaller than zero; supplementary material, table S13). Different letters at the top of the panels denote significant differences in LFCs among the four temperature-responsive groups in each treatment (Tukey's HSD, *p*_adj_ < 0.05; supplementary material, table S14).

## 4. Discussion

Temperature transition is a unique component of fluctuating temperatures compared to constant temperatures [18–21]. In our study, alongside identifying species that preferred constant high (H-superior species) or low (L-superior species) temperatures using the same methods as previous studies [29,48], we also identified species that specifically responded to temperature transitions (RF-superior and -inferior species). We found that both H- and L-superior species primarily responded to the component high and low temperatures and were insensitive to temperature transitions. Specifically, their performances at all fluctuating temperatures were roughly equal to their time-averaged performances at the component high and low temperatures (figure 3). By contrast, both RF-superior and -inferior species exhibited significant deviations from their time-averaged performances under rapid temperature fluctuations (figure 3). It is worth noting that the RF-responsive species were more abundant than H- and L-superior species (figure 2), and the community divergence between the RF treatment and all other treatments primarily stemmed from species sensitive to temperature transitions. Our findings highlight that soil bacteria sensitive to temperature transitions constitute a significant proportion in natural communities, and their specific responses to temperature transitions play a crucial role in community dynamics at fluctuating temperatures.

Furthermore, our study suggests that the RF-superior species gain competitive advantages primarily due to temperature transitions, as these advantages only emerged when they experienced frequent temperature transitions (23 times in the IF treatment and 239 times in the HF treatment). Previous selection experiments have implied that bacteria can specifically adapt to temperature transitions [19,20,22]; our study further implies that this strategy may be widely adopted by soil bacteria. In addition to RF-superior species, we also identified RF-inferior species that were sensitive to temperature transitions and maladapted under rapid temperature fluctuations. However, they showed better performances than RF-superior species at constant temperatures (figure 5). Similarly, a previous study of selection experiment has also found that the evolved lines specifically adapting to fluctuations in temperature exhibit poorer performances at all constant temperatures compared to those evolved at constant temperatures [19]. Accordingly, we speculate that for soil bacteria, actively responding to temperature transitions may incur universal fitness costs under constant temperature conditions.

Although the influence of temperature transitions was minimized under slow temperature fluctuation, significant deviations in responses of bacterial species to slow temperature fluctuation from those at medium temperature were still observed (figure 1; supplementary material, figure S2, table S5). Alongside a previous study on soil-bacterial community [29], our study validates that when the frequency of temperature fluctuation is low, the effects of fluctuating temperatures can be roughly approximated as the time-averaged effects of high and low temperatures (figure 3). Under such circumstances, the equivalence of fluctuating temperature with low frequency and medium temperature in determining the temperature responses of soil bacteria requires that the changes in bacterial abundance should increase linearly with temperature [24–26]. However, we found that the changes in bacterial abundance showed an accelerating increase as temperature increased (figure 4). The consequence of such nonlinearity was that the changes in bacterial abundance at medium temperature were not equal to the time-averaged changes in bacterial abundance, which were the approximate bacterial abundance changes under slow temperature fluctuation. In summary, our results indicate that the nonlinearity of thermal performance across temperature is another force driving differentiation of species’ temperature responses between fluctuating and medium temperatures.

Theoretically, both varying bacterial thermal traits and interspecific interactions can lead bacteria to exhibit distinct responses to different temperature regimes in natural communities [2,5,31,49]. Moreover, biotic interactions can substantially modify the temperature responses of focal species and generally make species performance difficult to predict, potentially obscuring or weakening the observable effects of temperature [2,31,50]. This contrasts with our observation that species performance under slow temperature fluctuations could be well predicted based on their performance under constant high and low temperatures. In addition, studies focusing on the size and spatial distribution of soil-bacterial communities at the microscale have provided solid evidence that the distribution of bacterial communities in soil is highly patchy [51–53]. Their results have shown that soil bacteria reside in numerous small communities, 90% of which contain fewer than 100 single-species cells, and that the interaction range of soil bacteria is very limited [52–55]. These lines of evidence together suggest that the distinct responses to different temperature regimes observed in our study are more likely to be attributable to varying bacterial thermal traits than to interspecific interactions. Furthermore, it is worth noting that the thermal traits of soil bacteria may depend in part on their life-history strategies and carbon substrate utilization [5,45]. We found that RF-superior species were more likely to be copiotrophs (supplementary material, table S9). Copiotrophs (*r*-strategists) have high growth rates and utilize labile carbon [44,56], which may reduce delays in achieving steady-state growth rates at new temperatures and enable fast responses to temperature transitions [57], thereby conferring competitive advantages under rapid temperature fluctuations.

There are four caveats to note about our experiment. Firstly, the temperature settings did not capture the entire temperature range of the sampling site (supplementary material, table S1). Previous studies with larger temperature differences (28°C versus 12°C) have identified more temperature-responsive species [29,48]. Moreover, given the current sequencing coverage, it remains challenging to determine whether a species absent from a given sample has gone extinct or simply falls below the detection threshold in natural soil-bacterial communities. Therefore, we cannot distinguish whether species loss at fluctuating temperatures was driven by the high-temperature phases or by a unique effect of temperature fluctuation per se. In general, the number of temperature-responsive species is underestimated in our study. Secondly, it should also be noted that soil communities from temperate climates (as in our study) are more likely to show adaptation to temperature transitions than tropical soil communities, which are exposed to smaller temperature variation. Thirdly, the soil-bacterial communities had not reached their stable states in our four-month experiment. Despite the accumulating evidence suggesting that the transient states of soil-bacterial communities might be prevalent [58–60], it is essential to investigate the communities at stable states under different temperature regimes. Finally, we oversimplified fluctuating temperatures as alternating high and low temperature phases in our experimental design. Further studies incorporating more levels of temperature settings with longer experimental duration and more realistic fluctuations in temperature will provide deeper insights into soil-bacterial community dynamics in a natural thermal environment.

## 5. Conclusion

Our work aims to elucidate how bacterial species respond to rapid and slow temperature fluctuations in natural communities. Previous studies have primarily focused on soil-bacterial species’ performances and community compositional changes across a gradient of constant temperatures [29,48,61], or have concentrated specifically on the temperature variance (i.e. the amplitudes of fluctuation) within the context of global warming [15–17]. As a supplement, our study highlights the importance of temperature transition and reveals the effects of frequency of temperature fluctuation on microbial performances and community dynamics. Specifically, we identified bacterial species that were uniquely responsive to frequent temperature transitions. We further demonstrated that two main processes operated simultaneously, leading to discrepancies among soil-bacterial species in response to fluctuating temperatures and medium temperature, that is (i) the bacterial-specific responses to temperature transition, (ii) the nonlinearity of bacterial performances across temperatures. In general, our findings based on highly simplified fluctuation regimes with three distinct frequencies indicate that temperature fluctuations at different temporal scales, ranging from diurnal to longer-term cycles, might each exert profound and distinctive influences on microbial communities in nature. Our results also suggest that mean temperature alone is an inadequate surrogate variable for capturing the complexities of fluctuating temperatures in microbial community analyses.

## Supplementary Material



## Data Availability

All raw sequence data generated by this study have been made available in the NCBI Sequence Read Archive under BioProject PRJNA1233643. Metadata are also stored in the SRA BioProject PRJNA1233643. The R script and data used for the analyses in this study have been deposited in an OSF repository [62]. Supplementary material is available online [63].
