# Supplementary material for: Ecological responses of soil bacteria to rapid and slow temperature fluctuations
Source: Proc Biol Sci. 2026 Oct 7;293(2080):20261267. doi: 10.1098/rspb.2026.1267 (PMC13641917; doi:10.1098/rspb.2026.1267)
Supplement: Supplementary file 1 [file rspb.2026.1267.zip › rspb.2026.1267/rspb20261267_si_001.docx]

**Electronic supplementary material**

**Ecological responses of soil bacteria to rapid and slow temperature fluctuations**

Xin-Feng Zhao, Yu-Chen Qiu, Lin Li, Chang Yang, Xu Zheng, Wen-Sheng Shu, Yi-Qi Hao*

**Authors’ institutions:** Guangzhou Key Laboratory of Subtropical Biodiversity and Biomonitoring, Guangdong Provincial Key Laboratory of Biotechnology for Plant Development, School of Life Sciences, South China Normal University, Guangzhou 510631, People's Republic of China

*** Corresponding author:** Yi-Qi Hao

**E-mail address:** [yiqihao@m.scnu.edu.cn](mailto:yiqihao@m.scnu.edu.cn)

**Journal:** *Proceedings of the Royal Society B: Biological Sciences*
**Article DOI:** 10.1098/rspb.2026.1267

**Contents of this File:**

Supplementary Figure 1 - Figure 5,

Supplementary Table 1 - Table 14,

Supplementary Method S1, S2,

and Supplementary References.

**Figure S1** **The copy number of 16S rRNA genes per gram of soil in different treatments over time.** Quantitative PCR was performed with 80 soil samples (n = 8 for original soil at T0, and n = 4 for soil samples at each of the following three time points in each treatment, Method S2). There were no significant differences between the temperature treatments and time points (Table S2).

**Figure S2 Metric multidimensional scaling ordination of soil bacterial communities at four time points by principal coordinate analysis (PCoA).** Each point represents an experimental soil bacterial community (n = 48 for each time point). Distances among communities represent Bray-Curtis dissimilarities. The communities are coloured based on the treatments. The composition of communities in different treatments was significantly different from each other at all time points except T0 (Table S3). Furthermore, soil bacterial communities had diverged into two groups in our experiment since T1, one group consisted of three constant temperature treatments and the LF treatment (95% normal confidence ellipses, blue circle), while the other group consisted of the IF and HF treatments with higher temperature fluctuation frequencies (95% normal confidence ellipses, pink circle).

**Figure S3** **A schematic illustration of the procedure of identifying temperature-responsive species.** Firstly, we compared the abundance of each common species between the H and L treatments at the end of the experiment (measured as Log2-fold changes, LFCs), and identified H- and L-superior species as those that had significantly higher abundance at the high or low temperature, respectively. Posterior examination confirmed that H-/L-superior species only responded to constant temperatures, i.e., temperature transitions exerted little effects on their performances (Figure 3; Table S10, S11). Secondly, we compared the abundance of the rest of common species in each of the three fluctuating temperature treatments with that in the H or L treatment, and identified species responding to fluctuating temperatures as those showed significant differences in abundance between fluctuating temperature treatment (LF, IF or HF treatment) and constant temperature treatment (H or L treatment) (measured as LFCs). For instance, species which had significantly higher abundance in the LF treatment than in the H and/or L treatments were defined as LF-superior species (IF-superior species for the IF treatment, and HF-superior species for the HF treatment); meanwhile, species which had significantly lower abundance in the LF treatment than in the H and/or L treatments were defined as LF-inferior species (IF-inferior species for the IF treatment, and HF-inferior species for the HF treatment). The flowchart has included all the situations in our study.

**Figure S4. Changes in species richness of soil bacterial communities in the six treatments over time.** Lines indicate fitted linear relationships between species richness and time; solid lines denote slopes significantly different from zero, and dashed lines denote slopes not significantly different from zero. Statistics for the linear regressions are shown in each panel.

**Figure S5 Hierarchical clustering of experimental communities at T1 (a), T2 (b) and T3 (c), respectively.** Ward’s minimum variance clustering method was used, and communities from different treatments were denoted by different colours.

Table S1 Monthly mean temperature, monthly mean minimum/maximum temperatures and monthly extreme minimum/maximum temperatures of the sampling site. The data is based on weather records from 1971 to 2000 and is obtained from the website of Weather China ([www.weather.com.cn](http://www.weather.com.cn)).

| Month | Monthly mean temperature | Monthly mean minimum temperature | Monthly mean maximum temperature | Monthly extreme minimum temperature | Monthly extreme maximum temperature |
| --- | --- | --- | --- | --- | --- |
| 1 | -13.5 | -18.5 | -7.5 | -32.3 | 5.5 |
| 2 | -9.3 | -14.8 | -3.1 | -29 | 16.1 |
| 3 | -0.7 | -6.4 | 5 | -24.9 | 19.8 |
| 4 | 9 | 2.8 | 15.3 | -10.5 | 29.2 |
| 5 | 16 | 9.9 | 22.2 | -3 | 35 |
| 6 | 21.1 | 15.9 | 26.7 | 5 | 37.3 |
| 7 | 23.7 | 19.6 | 28.2 | 12 | 35.2 |
| 8 | 22.2 | 17.8 | 27.3 | 6.6 | 34.2 |
| 9 | 16 | 10.5 | 22.4 | -2.9 | 31.8 |
| 10 | 7.7 | 2.3 | 14.3 | -13.2 | 27.7 |
| 11 | -2 | -6.6 | 3.5 | -21.8 | 20.4 |
| 12 | -10 | -14.7 | -4.5 | -29.9 | 12.2 |

Table S2 Summary of ANOVA statistics of the time and treatment effects on the log-transformed copy number of 16S rRNA genes per gram of soil.

|  | df | Sum of squares | Mean of squares | *F* | *p* |
| --- | --- | --- | --- | --- | --- |
| Treatment | 6 | 0.0060 | 0.0010 | 0.1441 | 0.989 |
| Time | 2 | 0.0119 | 0.0059 | 0.8617 | 0.427 |
| Residuals | 71 | 0.4895 | 0.0069 |  |  |

Table S3 Summary of permutation MANOVA statistics for the treatment effects on soil bacterial communities at each time point (permutations=9999).

|  |  | df | Sum of squares | Mean of squares | *F* | *R^2^* | *p* |
| --- | --- | --- | --- | --- | --- | --- | --- |
| T0 | Treatment | 5 | 0.222 | 0.044 | 0.941 | 0.103 | 0.9328 |
|  | Residuals | 41 | 1.933 | 0.047 |  | 0.897 |  |
|  | Total | 46 | 2.154 |  |  | 1.000 |  |
| T1 | Treatment | 5 | 0.421 | 0.084 | 2.059 | 0.197 | 0.0001 |
|  | Residuals | 42 | 1.716 | 0.041 |  | 0.803 |  |
|  | Total | 47 | 2.137 |  |  | 1.000 |  |
| T2 | Treatment | 5 | 0.445 | 0.089 | 2.120 | 0.202 | 0.0001 |
|  | Residuals | 42 | 1.764 | 0.042 |  | 0.798 |  |
|  | Total | 47 | 2.209 |  |  | 1.000 |  |
| T3 | Treatment | 5 | 0.528 | 0.106 | 2.304 | 0.215 | 0.0001 |
|  | Residuals | 42 | 1.925 | 0.046 |  | 0.785 |  |
|  | Total | 47 | 2.453 |  |  | 1.000 |  |

Table S4 Summary of permutation MANOVA statistics for the treatment and time effects on soil bacterial communities (permutations=9999).

|  | df | Sum of squares | Mean of squares | *F* | *R^2^* | *p* |
| --- | --- | --- | --- | --- | --- | --- |
| Treatment | 5 | 0.700 | 0.140 | 3.188 | 0.070 | 0.0001 |
| Time | 3 | 0.986 | 0.329 | 7.478 | 0.099 | 0.0001 |
| Treatment:Time | 15 | 0.916 | 0.061 | 1.390 | 0.092 | 0.0001 |
| Residuals | 167 | 7.337 | 0.044 |  | 0.738 |  |
| Total | 190 | 9.939 |  |  | 1.000 |  |

Table S5 Summary of the permutation MANOVA statistics for the pairwise comparisons of temperature effects on soil bacterial communities at the end time point (permutations=9999, *p* values were adjusted following the Benjamini-Hochberg procedure).

|  |  | | df | | Sum of squares | | Mean of squares | | *F* | | *R^2^* | | *p* | | *p_adj_* | |  |
| --- | --- | --- | --- | --- | --- | --- | --- | --- | --- | --- | --- | --- | --- | --- | --- | --- | --- |
| H vs. L | Treatment | | 1 | | 0.14758 | | 0.147577 | | 3.234 | | 0.18765 | | 0.0002 | | 0.00056 | |  |
|  | Residuals | | 14 | | 0.63887 | | 0.045634 | | 0.81235 | |  | |  | |  | |  |
|  | Total | | 15 | | 0.78645 | | 1 | |  | |  | |  | |  | |  |
| H vs. M | Treatment | | 1 | | 0.11863 | | 0.11863 | | 2.6385 | | 0.15858 | | 0.0005 | | 0.00083 | |  |
|  | Residuals | | 14 | | 0.62944 | | 0.04496 | | 0.84142 | |  | |  | |  | |  |
|  | Total | | 15 | | 0.74807 | | 1 | |  | |  | |  | |  | |  |
| H vs. LF | Treatment | | 1 | | 0.1058 | | 0.105802 | | 2.4154 | | 0.14714 | | 0.0008 | | 0.00109 | |  |
|  | Residuals | | 14 | | 0.61326 | | 0.043804 | | 0.85286 | |  | |  | |  | |  |
|  | Total | | 15 | | 0.71906 | | 1 | |  | |  | |  | |  | |  |
| H vs. IF | Treatment | | 1 | | 0.14591 | | 0.145911 | | 3.1549 | | 0.18391 | | 0.0002 | | 0.00056 | |  |
|  | Residuals | | 14 | | 0.64749 | | 0.046249 | | 0.81609 | |  | |  | |  | |  |
|  | Total | | 15 | | 0.7934 | | 1 | |  | |  | |  | |  | |  |
| H vs. HF | Treatment | | 1 | | 0.1516 | | 0.151601 | | 2.948 | | 0.17394 | | 0.0003 | | 0.00056 | |  |
|  | Residuals | | 14 | | 0.71994 | | 0.051425 | | 0.82606 | |  | |  | |  | |  |
|  | Total | | 15 | | 0.87155 | | 1 | |  | |  | |  | |  | |  |
| L vs. M | Treatment | | 1 | | 0.05933 | | 0.059325 | | 1.3702 | | 0.08915 | | 0.0072 | | 0.00771 | |  |
|  | Residuals | | 14 | | 0.60613 | | 0.043295 | | 0.91085 | |  | |  | |  | |  |
|  | Total | | 15 | | 0.66546 | | 1 | |  | |  | |  | |  | |  |
| L vs. LF | Treatment | | 1 | | 0.06235 | | 0.062354 | | 1.4797 | | 0.09559 | | 0.0039 | | 0.00450 | |  |
|  | Residuals | | 14 | | 0.58995 | | 0.042139 | | 0.90441 | |  | |  | |  | |  |
|  | Total | | 15 | | 0.6523 | | 1 | |  | |  | |  | |  | |  |
| L vs. IF | Treatment | | 1 | | 0.11036 | | 0.110361 | | 2.4753 | | 0.15024 | | 0.0003 | | 0.00056 | |  |
|  | Residuals | | 14 | | 0.62418 | | 0.044584 | | 0.84976 | |  | |  | |  | |  |
|  | Total | | 15 | | 0.73454 | | 1 | |  | |  | |  | |  | |  |
| L vs. HF | Treatment | | 1 | | 0.11105 | | 0.11105 | | 2.2317 | | 0.13749 | | 0.0006 | | 0.00090 | |  |
|  | Residuals | | 14 | | 0.69663 | | 0.04976 | | 0.86251 | |  | |  | |  | |  |
|  | Total | | 15 | | 0.80768 | | 1 | |  | |  | |  | |  | |  |
| M vs. LF | Treatment | | 1 | | 0.06636 | | 0.066359 | | 1.6003 | | 0.10258 | | 0.0013 | | 0.00163 | |  |
|  | Residuals | | 14 | | 0.58052 | | 0.041466 | | 0.89742 | |  | |  | |  | |  |
|  | Total | | 15 | | 0.64688 | | 1 | |  | |  | |  | |  | |  |
| M vs. IF | Treatment | | 1 | | 0.09202 | | 0.092023 | | 2.0957 | | 0.1302 | | 0.0003 | | 0.00056 | |  |
|  | Residuals | | 14 | | 0.61475 | | 0.043911 | | 0.8698 | |  | |  | |  | |  |
|  | Total | | 15 | | 0.70677 | | 1 | |  | |  | |  | |  | |  |
| M vs. HF | Treatment | | 1 | | 0.09892 | | 0.098919 | | 2.0152 | | 0.12583 | | 0.0003 | | 0.00056 | |  |
|  | Residuals | | 14 | | 0.68721 | | 0.049086 | | 0.87417 | |  | |  | |  | |  |
|  | Total | | 15 | | 0.78613 | | 1 | |  | |  | |  | |  | |  |
|  | |  | | df | | Sum of squares | | Mean of squares | | *F* | | *R^2^* | | *p* | | *p_adj_* | |
| LF vs. IF | | Treatment | | 1 | | 0.12301 | | 0.123011 | | 2.8771 | | 0.17048 | | 0.0001 | | 0.00056 | |
|  | | Residuals | | 14 | | 0.59856 | | 0.042754 | | 0.82952 | |  | |  | |  | |
|  | | Total | | 15 | | 0.72157 | | 1 | |  | |  | |  | |  | |
| LF vs. HF | | Treatment | | 1 | | 0.13074 | | 0.13074 | | 2.7278 | | 0.16307 | | 0.0001 | | 0.00056 | |
|  | | Residuals | | 14 | | 0.67102 | | 0.04793 | | 0.83693 | |  | |  | |  | |
|  | | Total | | 15 | | 0.80176 | | 1 | |  | |  | |  | |  | |
| IF vs. HF | | Treatment | | 1 | | 0.05995 | | 0.059949 | | 1.1901 | | 0.07834 | | 0.1066 | | 0.10660 | |
|  | | Residuals | | 14 | | 0.70525 | | 0.050375 | | 0.92166 | |  | |  | |  | |
|  | | Total | | 15 | | 0.7652 | | 1 | |  | |  | |  | |  | |

Table S6 Summary of ANOVA statistics and Tukey’s multiple comparisons of species richness between the six treatments at the end of the experiment.

Table S6-1 ANOVA statistics of species richness between the six treatments at the end of the experiment.

|  | df | Sum of squares | Mean of squares | *F* | *p* |
| --- | --- | --- | --- | --- | --- |
| Treatment | 5 | 746817 | 149363 | 8.538 | 0.00001 |
| Residuals | 42 | 734751 | 17494 |  |  |

Table S6-2 Tukey’s multiple comparisons of species richness between treatment pairs at the end of the experiment.

| Contrasts | Difference in means | Lower end of 95% confidence interval | Upper end of 95% confidence interval | *p*_adj_ |
| --- | --- | --- | --- | --- |
| M vs. L | 4.375 | -193.0472 | 201.7972 | 1.00000 |
| H vs. L | -364.375 | -561.7972 | -166.9528 | 0.00003 |
| LF vs. L | -157.375 | -354.7972 | 40.0472 | 0.18674 |
| IF vs. L | -168.25 | -365.6722 | 29.1722 | 0.13459 |
| HF vs. L | -179.625 | -377.0472 | 17.7972 | 0.09317 |
| H vs. M | -368.75 | -566.1722 | -171.3278 | 0.00002 |
| LF vs. M | -161.75 | -359.1722 | 35.6722 | 0.16418 |
| IF vs. M | -172.625 | -370.0472 | 24.7972 | 0.11718 |
| HF vs. M | -184 | -381.4222 | 13.4222 | 0.08036 |
| LF vs. H | 207 | 9.5778 | 404.4222 | 0.03502 |
| IF vs. H | 196.125 | -1.2972 | 393.5472 | 0.05241 |
| HF vs. H | 184.75 | -12.6722 | 382.1722 | 0.07832 |
| IF vs. LF | -10.875 | -208.2972 | 186.5472 | 0.99998 |
| HF vs. LF | -22.25 | -219.6722 | 175.1722 | 0.99939 |
| HF vs. IF | -11.375 | -208.7972 | 186.0472 | 0.99998 |

Table S7 Pagel's tests of phylogenetic conservatism of soil bacterial responses to different temperatures.

|  | lambda | logL(lambda) | LR(lambda=0) | *p* |
| --- | --- | --- | --- | --- |
| H-superior species | 0.0066 | 5872.08 | 1.577 | 0.2091 |
| L-superior species | 0.011 | 5879.95 | 12.210 | 0.0005 |
| RF-superior species | 0.0461 | 4348.43 | 112.220 | <0.0001 |
| RF-inferior species | 0.0434 | 4478.32 | 57.052 | <0.0001 |

Table S8 Statistical tests of whether the bacterial species belonging to a particular temperature-responsive group are more phylogenetically related to one another than to other species in the soil bacterial communities (permutation = 9999). NRI indicates how temperature-responsive species belonging to same group cluster across the whole phylogenetic tree, while NTI indicates how they cluster at the tips of the phylogenetic tree.

Table S8-1 NRI test

|  | ntaxa | mpd.obs | mpd.rand.mean | mpd.rand.sd | mpd.obs.z | NRI | mpd.obs.p |
| --- | --- | --- | --- | --- | --- | --- | --- |
| H-superior species | 45 | 0.67 | 0.76 | 0.033 | -2.83 | 2.83 | 0.0029 |
| L-superior species | 49 | 0.77 | 0.76 | 0.032 | 0.18 | -0.18 | 0.5687 |
| RF-superior species | 90 | 0.77 | 0.76 | 0.023 | 0.55 | -0.55 | 0.7043 |
| RF-inferior species | 72 | 0.65 | 0.76 | 0.026 | -4.28 | 4.28 | 0.0001 |

Table S8-2 NTI test

|  | ntaxa | mntd.obs | mntd.rand.mean | mntd.rand.sd | mntd.obs.z | NTI | mntd.obs.p |
| --- | --- | --- | --- | --- | --- | --- | --- |
| H-superior species | 45 | 0.17 | 0.3 | 0.029 | -4.39 | 4.39 | 0.0001 |
| L-superior species | 49 | 0.22 | 0.29 | 0.027 | -2.6 | 2.6 | 0.0046 |
| RF-superior species | 90 | 0.14 | 0.23 | 0.018 | -5.34 | 5.34 | 0.0001 |
| RF-inferior species | 72 | 0.15 | 0.25 | 0.021 | -4.67 | 4.67 | 0.0001 |

Table S9 Summary of the number of copiotrophic and oligotrophic species in each of the four temperature-responsive groups, and Chi-square tests of whether the copiotroph/oligotroph ratio significantly deviated from 1 in each group. Copiotrophic and oligotrophic lifestyle were classified based on the phylum of each bacterial species (Fierer et al., 2007; Ho et al., 2017; Singh, 2018; Chen et al., 2022). Specifically, *α-Proteobacteria*, *β-Proteobacteria*, *γ-Proteobacteria*, *Bacteroidetes* and *Firmicutes* were designated as copiotrophic lifestyle; whereas *δ-Proteobacteria*, *Acidobacteria*, *Actinobacteria*, *Verrucomicrobia*, *Gemmatimonadetes* were designated as oligotrophic lifestyle.

|  | The number of species in different lifestyle groups | | | Copiotroph/oligotroph ratio  and Chi-square tests | | | |
| --- | --- | --- | --- | --- | --- | --- | --- |
|  | Copiotroph | Oligotroph | Unclassified | C/O ratio | χ^2^ | df | p values |
| H-superior | 18 | 25 | 2 | 0.72 | 1.14 | 1 | 0.29 |
| L-superior | 24 | 24 | 1 | 1 | 0 | 1 | 1 |
| RF-superior | 59 | 25 | 6 | 2.36 | 13.76 | 1 | 0.0002 |
| RF-inferior | 27 | 38 | 7 | 0.71 | 1.86 | 1 | 0.17 |

Table S10 Summary statistics of testing whether the observed LFCs of four groups of temperature-responsive species in the three fluctuating temperature treatments are significantly different from the predicted LFCs (the time-averaged LFCs or LFCs in the M treatment). The mixed-effects linear models were fitted separately for H-superior, L-superior, RF-superior and RF-inferior species using the lme function in the 'nlme' R package, with LFC category (observed vs. predicted) as a fixed factor and species identity as a random factor.

Table S10-1 The time-averaged LFCs as predictor

|  |  | Value | Std.error | df | *t*-value | *p* value |
| --- | --- | --- | --- | --- | --- | --- |
| H-superior species | LF | 0.282 | 0.160 | 132 | 1.767 | 0.0796 |
|  | IF | 0.154 | 0.160 | 132 | 0.965 | 0.3365 |
|  | HF | -0.024 | 0.160 | 132 | -0.152 | 0.8793 |
| L-superior species | LF | 0.253 | 0.176 | 144 | 1.434 | 0.1537 |
|  | IF | 0.308 | 0.176 | 144 | 1.746 | 0.0829 |
|  | HF | 0.165 | 0.176 | 144 | 0.935 | 0.3513 |
| RF-superior species | LF | 0.061 | 0.102 | 267 | 0.603 | 0.547 |
|  | IF | 1.126 | 0.102 | 267 | 11.075 | <0.0001 |
|  | HF | 0.750 | 0.102 | 267 | 7.372 | <0.0001 |
| RF- inferior species | LF | 0.067 | 0.116 | 213 | 0.578 | 0.564 |
|  | IF | -1.045 | 0.116 | 213 | -9.023 | <0.0001 |
|  | HF | -1.059 | 0.116 | 213 | -9.149 | <0.0001 |

Table S10-2 The LFCs at the medium temperature as predictor

|  |  | Value | Std.error | df | *t*-value | *p* value |
| --- | --- | --- | --- | --- | --- | --- |
| H-superior species | LF | 0.441 | 0.187 | 132 | 2.361 | 0.0197 |
|  | IF | 0.313 | 0.187 | 132 | 1.676 | 0.0961 |
|  | HF | 0.135 | 0.187 | 132 | 0.722 | 0.4713 |
| L-superior species | LF | -0.562 | 0.181 | 144 | -3.106 | 0.0023 |
|  | IF | -0.507 | 0.181 | 144 | -2.802 | 0.0058 |
|  | HF | -0.650 | 0.181 | 144 | -3.592 | 0.0004 |
| RF-superior species | LF | -0.264 | 0.101 | 267 | -2.606 | 0.0097 |
|  | IF | 0.801 | 0.101 | 267 | 7.906 | <0.0001 |
|  | HF | 0.424 | 0.101 | 267 | 4.189 | <0.0001 |
| RF- inferior species | LF | 0.164 | 0.124 | 213 | 1.320 | 0.1882 |
|  | IF | -0.948 | 0.124 | 213 | -7.640 | <0.0001 |
|  | HF | -0.962 | 0.124 | 213 | -7.757 | <0.0001 |

Table S11 Summary statistics of linear relationships between the predicted and observed LFCs of temperature-responsive species in the three fluctuating temperature treatments. The linear regressions were fitted separately for the H-/L-superior species and RF-superior/-inferior species.

Table S11-1 The time-averaged performance as the predictor.

|  |  |  | Estimate | Std. Error | *t* | *p* | *R^2^* | *adj R^2^* |
| --- | --- | --- | --- | --- | --- | --- | --- | --- |
| H-/L-superior species | LF | Intercept | 0.27 | 0.091 | 2.955 | 0.00397 | 0.735 | 0.732 |
|  |  | Slope | 0.947 | 0.059 | 15.976 | <0.0001 |  |  |
|  | IF | Intercept | 0.252 | 0.106 | 2.372 | 0.0198 | 0.549 | 0.544 |
|  |  | Slope | 0.726 | 0.069 | 10.571 | <0.0001 |  |  |
|  | HF | Intercept | 0.088 | 0.119 | 0.738 | 0.463 | 0.526 | 0.521 |
|  |  | Slope | 0.783 | 0.077 | 10.112 | <0.0001 |  |  |
| RF-superior/ RF-inferior species | LF | Intercept | 0.037 | 0.047 | 0.78 | 0.437 | 0.728 | 0.727 |
|  |  | Slope | 0.855 | 0.041 | 20.72 | <0.0001 |  |  |
|  | IF | Intercept | -0.004 | 0.094 | -0.045 | 0.964 | 0.012 | 0.006 |
|  |  | Slope | 0.117 | 0.083 | 1.414 | 0.159 |  |  |
|  | HF | Intercept | -0.131 | 0.088 | -1.491 | 0.138 | 0.267 | 0.262 |
|  |  | Slope | 0.59 | 0.077 | 7.624 | <0.0001 |  |  |

Table S11-2 The performance at the medium temperature as the predictor

|  |  |  | Estimate | Std. Error | *t* | *p* | *R^2^* | *adj R^2^* |
| --- | --- | --- | --- | --- | --- | --- | --- | --- |
| H-/L-superior species | LF | Intercept | -0.036 | 0.141 | -0.251 | 0.802 | 0.428 | 0.421 |
|  |  | Slope | 0.888 | 0.107 | 8.29 | <0.0001 |  |  |
|  | IF | Intercept | 0.004 | 0.134 | 0.033 | 0.974 | 0.348 | 0.341 |
|  |  | Slope | 0.711 | 0.101 | 7.012 | <0.0001 |  |  |
|  | HF | Intercept | -0.154 | 0.154 | -0.996 | 0.322 | 0.285 | 0.277 |
|  |  | Slope | 0.707 | 0.117 | 6.048 | <0.0001 |  |  |
| RF-superior/ RF-inferior species | LF | Intercept | -0.079 | 0.053 | -1.509 | 0.133 | 0.65 | 0.648 |
|  |  | Slope | 0.888 | 0.052 | 17.228 | <0.0001 |  |  |
|  | IF | Intercept | -0.018 | 0.093 | -0.189 | 0.85 | 0.022 | 0.016 |
|  |  | Slope | 0.174 | 0.091 | 1.923 | 0.056 |  |  |
|  | HF | Intercept | -0.208 | 0.085 | -2.435 | 0.016 | 0.292 | 0.287 |
|  |  | Slope | 0.678 | 0.084 | 8.115 | <0.0001 |  |  |

Table S12 Summary statistics of the treatment effects on the log2-fold changes across the whole experiment of bacterial species in our experiment. The mixed-effects linear models were fitted separately for species with increased abundance (n=219) and species with decreased abundance (n=238), using the lme function in the ‘nlme’ R package, with temperature treatment as a fixed factor and species identity as a random factor.

|  |  | Value | Std.error | df | *t*-value | *p* value |
| --- | --- | --- | --- | --- | --- | --- |
| Species with increased abundance (n=219) | M vs. L | 0.2017 | 0.0774 | 872 | 2.604 | 0.0094 |
|  | H vs. L | 0.5366 | 0.0774 | 872 | 6.929 | <0.0001 |
|  | LF vs. L | 0.3345 | 0.0774 | 872 | 4.319 | <0.0001 |
|  | Average vs. L | 0.2683 | 0.0774 | 872 | 3.465 | 0.0006 |
|  | H vs. M | 0.3349 | 0.0774 | 872 | 4.325 | <0.0001 |
|  | LF vs. M | 0.1328 | 0.0774 | 872 | 1.715 | 0.0867 |
|  | Average vs. M | 0.0666 | 0.0774 | 872 | 0.860 | 0.3899 |
|  | LF vs. H | -0.2021 | 0.0774 | 872 | -2.610 | 0.0092 |
|  | Average vs. H | -0.2683 | 0.0774 | 872 | -3.465 | 0.0006 |
|  | Average vs. LF | -0.0662 | 0.0774 | 872 | -0.855 | 0.3929 |
| Species with decreased abundance (n=238) | M vs. L | -0.0081 | 0.0695 | 948 | -0.117 | 0.9068 |
|  | H vs. L | -0.9104 | 0.0695 | 948 | -13.104 | <0.0001 |
|  | LF vs. L | -0.3186 | 0.0695 | 948 | -4.586 | <0.0001 |
|  | Average vs. L | -0.4552 | 0.0695 | 948 | -6.552 | <0.0001 |
|  | H vs. M | -0.9022 | 0.0695 | 948 | -12.986 | <0.0001 |
|  | LF vs. M | -0.3105 | 0.0695 | 948 | -4.469 | <0.0001 |
|  | Average vs. M | -0.4470 | 0.0695 | 948 | -6.435 | <0.0001 |
|  | LF vs. H | 0.5917 | 0.0695 | 948 | 8.517 | <0.0001 |
|  | Average vs. H | 0.4552 | 0.0695 | 948 | 6.552 | <0.0001 |
|  | Average vs. LF | -0.1366 | 0.0695 | 948 | -1.966 | 0.0496 |

Table S13 Summary of one-sample t-test of differences between zero and the log2-fold changes of H-superior, L-superior, RF-superior and FR-inferior species across the whole experiment in each temperature treatment.

| Group | Treatment | N | mean | sd | *t* | *p* | *p_adj_* |
| --- | --- | --- | --- | --- | --- | --- | --- |
| H-superior species | L | 45 | -0.227 | 1.047 | -1.453 | 0.1530 | 0.1669 |
| L-superior species | L | 49 | 0.475 | 1.284 | 2.588 | 0.1270 | 0.1524 |
| RF-superior species | L | 90 | -0.388 | 1.173 | -3.142 | 0.0023 | 0.0042 |
| RF-inferior species | L | 72 | 0.385 | 1.013 | 3.224 | 0.0019 | 0.0038 |
| H-superior species | M | 45 | 0.769 | 1.227 | 4.207 | 0.0001 | 0.0003 |
| L-superior species | M | 49 | 0.083 | 1.213 | 0.481 | 0.6325 | 0.6325 |
| RF-superior species | M | 90 | -0.222 | 1.059 | -1.991 | 0.0495 | 0.0660 |
| RF-inferior species | M | 72 | 0.165 | 0.942 | 1.49 | 0.1407 | 0.1608 |
| H-superior species | H | 45 | 2.085 | 2.147 | 6.512 | 0.0000 | <0.0001 |
| L-superior species | H | 49 | -1.939 | 1.683 | -8.063 | 0.0000 | <0.0001 |
| RF-superior species | H | 90 | -0.707 | 1.376 | -4.872 | 0.0000 | <0.0001 |
| RF-inferior species | H | 72 | 0.14 | 1.15 | 1.03 | 0.3064 | 0.3197 |
| H-superior species | LF | 45 | 1.211 | 1.663 | 4.885 | 0.0000 | <0.0001 |
| L-superior species | LF | 49 | -0.479 | 1.322 | -2.537 | 0.0145 | 0.0218 |
| RF-superior species | LF | 90 | -0.486 | 1.099 | -4.196 | 0.0001 | 0.0002 |
| RF-inferior species | LF | 72 | 0.329 | 0.996 | 2.804 | 0.0065 | 0.0112 |
| H-superior species | IF | 45 | 1.083 | 1.407 | 5.164 | 0.0000 | <0.0001 |
| L-superior species | IF | 49 | -0.424 | 1.247 | -2.38 | 0.0213 | 0.0301 |
| RF-superior species | IF | 90 | 0.579 | 0.775 | 7.084 | 0.0000 | <0.0001 |
| RF-inferior species | IF | 72 | -0.782 | 1.186 | -5.599 | 0.0000 | <0.0001 |
| H-superior species | HF | 45 | 0.895 | 1.548 | 3.878 | 0.0003 | 0.0008 |
| L-superior species | HF | 49 | -0.557 | 1.436 | -2.719 | 0.0091 | 0.0146 |
| RF-superior species | HF | 90 | 0.204 | 1.144 | 1.694 | 0.0938 | 0.1184 |
| RF-inferior species | HF | 72 | -0.782 | 1.184 | -5.602 | 0.0000 | <0.0001 |

Table S14 Summary of ANOVA statistics of testing the differences in species' performances (log2-fold changes over time, LFCs) among four temperature-responsive groups (H-superior, L-superior, RF-superior, RF-inferior species) in each treatment, and the corresponding Tukey’s HSD tests of LFCs between the four groups.

Table S14-1 Summary of ANOVA statistics of the differences in LFCs of temperature-responsive groups in each temperature treatment.

|  |  | df | Sum of squares | Mean of squares | *F* | *p* |
| --- | --- | --- | --- | --- | --- | --- |
| L | Species group | 3 | 37.48 | 12.493 | 9.755 | <0.0001 |
|  | Residuals | 252 | 322.74 | 1.281 |  |  |
| M | Species group | 3 | 29.74 | 9.913 | 8.337 | <0.0001 |
|  | Residuals | 252 | 299.66 | 1.189 |  |  |
| H | Species group | 3 | 414.34 | 138.113 | 57.883 | <0.0001 |
|  | Residuals | 252 | 601.29 | 2.386 |  |  |
| LF | Species group | 3 | 105.83 | 35.276 | 23.177 | <0.0001 |
|  | Residuals | 252 | 383.54 | 1.522 |  |  |
| IF | Species group | 3 | 133.58 | 44.525 | 35.628 | <0.0001 |
|  | Residuals | 252 | 314.93 | 1.250 |  |  |
| HF | Species group | 3 | 99.28 | 33.095 | 19.150 | <0.0001 |
|  | Residuals | 252 | 435.5 | 1.728 |  |  |

Table S14-2 Summary of Tukey’s HSD tests of LFCs between the four temperature-responsive groups in each temperature treatment.

|  | Contrasts | Difference in means | Lower end of 95% confidence interval | | Upper end of 95% confidence interval | | *p*_adj_ | |
| --- | --- | --- | --- | --- | --- | --- | --- | --- |
| L | L-superior species vs. H-superior species | 0.702 | 0.097 | | 1.306 | | 0.0155 | |
|  | RF-superior species vs. H-superior species | -0.162 | -0.696 | | 0.373 | | 0.8623 | |
|  | RF-inferior species vs. H-superior species | 0.612 | 0.055 | | 1.168 | | 0.0247 | |
|  | RF-superior species vs. L-superior species | -0.863 | -1.383 | | -0.344 | | 0.0001 | |
|  | RF-inferior species vs. L-superior species | -0.090 | -0.632 | | 0.452 | | 0.9734 | |
|  | RF-inferior species vs. RF-superior species | 0.773 | 0.311 | | 1.236 | | 0.0001 | |
| M | L-superior species vs. H-superior species | -0.686 | -1.268 | | -0.104 | | 0.0135 | |
|  | RF-superior species vs. H-superior species | -0.992 | -1.507 | | -0.477 | | <0.0001 | |
|  | RF-inferior species vs. H-superior species | -0.604 | -1.140 | | -0.068 | | 0.0201 | |
|  | RF-superior species vs. L-superior species | -0.306 | -0.806 | | 0.195 | | 0.3927 | |
|  | RF-inferior species vs. L-superior species | 0.082 | -0.440 | | 0.604 | | 0.9773 | |
|  | RF-inferior species vs. RF-superior species | 0.388 | -0.058 | | 0.834 | | 0.1133 | |
| H | L-superior species vs. H-superior species | -4.023 | -4.848 | | -3.198 | | <0.0001 | |
|  | RF-superior species vs. H-superior species | -2.791 | -3.520 | | -2.062 | | <0.0001 | |
|  | RF-inferior species vs. H-superior species | -1.945 | -2.704 | | -1.186 | | <0.0001 | |
|  | RF-superior species vs. L-superior species | 1.232 | 0.523 | | 1.941 | | <0.0001 | |
|  | RF-inferior species vs. L-superior species | 2.078 | 1.339 | | 2.818 | | <0.0001 | |
|  | RF-inferior species vs. RF-superior species | 0.846 | 0.215 | | 1.478 | | 0.0035 | |
| LF | L-superior species vs. H-superior species | -1.690 | | -2.349 | -1.031 | <0.0001 | |  |
|  | RF-superior species vs. H-superior species | -1.697 | | -2.280 | -1.115 | <0.0001 | |  |
|  | RF-inferior species vs. H-superior species | -0.882 | | -1.488 | -0.275 | 0.0012 | |  |
|  | RF-superior species vs. L-superior species | -0.007 | | -0.574 | 0.559 | 1.0000 | |  |
|  | RF-inferior species vs. L-superior species | 0.808 | | 0.217 | 1.399 | 0.0027 | |  |
|  | RF-inferior species vs. RF-superior species | 0.815 | | 0.311 | 1.320 | 0.0002 | |  |
| IF | L-superior species vs. H-superior species | -1.507 | | -2.104 | -0.910 | <0.0001 | |  |
|  | RF-superior species vs. H-superior species | -0.504 | | -1.032 | 0.024 | 0.0671 | |  |
|  | RF-inferior species vs. H-superior species | -1.865 | | -2.415 | -1.316 | <0.0001 | |  |
|  | RF-superior species vs. L-superior species | 1.003 | | 0.489 | 1.516 | <0.0001 | |  |
|  | RF-inferior species vs. L-superior species | -0.358 | | -0.894 | 0.177 | 0.3097 | |  |
|  | RF-inferior species vs. RF-superior species | -1.361 | | -1.818 | -0.904 | <0.0001 | |  |
| HF | L-superior species vs. H-superior species | -1.472 | | -2.174 | -0.770 | <0.0001 | |  |
|  | RF-superior species vs. H-superior species | -0.702 | | -1.323 | -0.082 | 0.0194 | |  |
|  | RF-inferior species vs. H-superior species | -1.701 | | -2.348 | -1.055 | <0.0001 | |  |
|  | RF-superior species vs. L-superior species | 0.769 | | 0.166 | 1.373 | 0.0061 | |  |
|  | RF-inferior species vs. L-superior species | -0.230 | | -0.860 | 0.400 | 0.7809 | |  |
|  | RF-inferior species vs. RF-superior species | -0.999 | | -1.537 | -0.461 | <0.0001 | |  |

**Method S1: PCR amplification, high-throughput sequencing and amplicon analysis**

The V4 hypervariable regions of the 16S rRNA genes were amplified using the primer pair 515F (5'-GTGCCAGCMGCCGCGGTAA-3') and 806R (5'- CCGGACTACHVGGGTWTCTAAT-3') (Caporaso et al., 2011). The 50 µl PCR amplification reaction mixtures contained 25 µl Takara Premix Taq^TM^ (version 2.0), 1 µl (10 µM) of forward and reverse primers, 1 µl purified DNA template, and 22 µl nuclease-free water. The PCR amplification procedure consisted of an initial denaturing step at 94 °C for 3 min; 35 cycles of 94 °C for 45 s, 50 °C for 60 s, and 72 °C for 90 s; and a final extension of 10 min at 72 °C. Triplicate PCR products for each sample were pooled, concentrated and purified with QIAquick gel extraction kits (Qiagen, Chatsworth, CA). Equimolar amounts of the purified PCR products were pooled and then sequenced on a 2 × 250 bp paired-end Illumina NovaSeq platform (Illumina, San Diego, CA).

The raw sequencing data was processed using QIIME2 2019-07 (Bolyen et al., 2019). Briefly, data was first demultiplexed, primers and barcodes were removed, and paired-end reads were assembled. The *qiime quality-filter q-score-joined* was then applied to filter and trim the joined sequences based on quality scores and the presence of ambiguous nucleotide bases with the following parameters: *r* = 3, *q* = 4, *p* = 0.75, and *n* = 0 (Bokulich et al., 2013). Then, the sequences were denoised using the *qiime deblur denoise-16S* command with default parameters (Amir et al., 2017), and the lengths of all sequences were trimmed to 250 bp (where quality scores of 99% sequences were higher than 30 but began to drop rapidly). To obtain single-nucleotide resolution of taxonomic units, the Deblur algorithm was employed to identify amplicon sequence variants (ASVs), which were defined as “species” in our study (Callahan et al., 2017). *De novo* chimaera filtering was achieved with VSEARCH (parameters: dn = 0.000001, xn = 1000, minh = 10000000, mindiffs = 5), which was integrated within the *qiime deblur denoise-16S* pipeline. The phylogenetic tree was constructed with the SATé-enabled phylogenetic placement (SEPP) technique using the fragment-insertion plugin on Qiime2 platform (Janssen et al., 2018).

**Method S2: Real-time quantitative PCR**

We randomly chose eight replicates of the original soil bacterial communities at the beginning of experiment and four replicates in each treatment at each of the following time points (T1, T2, T3). Then, we evaluated the cell densities of bacteria in these soil microcosms by the copy number of the 16S rRNA gene per gram of soil. Real-time quantitative PCR (qPCR) was performed to estimate the copy number of the 16S rRNA gene per gram of soil with the primer set 515F/806R (V4 hypervariable regions: 5'-GTGCCAGCMGCCGCGGTAA-3' and 5'- CCGGACTACHVGGGTWTCTAAT-3'). The reactions were carried out in a QuantStudio5 real-time qPCR System (Applied Biosystems by Life Technologies, Singapore). Quantification was based on the increasing fluorescence intensity of the SYBR Green dye during amplification. The qPCR reactions were carried out in 20 μl volume, containing 0.5 μl of each 10-μM forward and reverse primers, 10 μl of PowerSYBR green PCR Master Mix (Applied Biosystems, USA), 7 μl of sterile DNA-free water and 2 μl of standard or soil DNA. QPCR consisted of an 8-min initial denaturation at 94°C, followed by 45 cycles of 40 s at 94°C, 40 s at 55.7°C, and 40 s at 72°C, and a final 10-min extension at 72°C. Standard curves were obtained using 10-fold serial dilution (10^3^-10^8^copies μl^-1^) of plasmids containing the same copy of a soil bacterial 16S rRNA gene. Proper dilution of soil DNA was chosen to minimize the qPCR inhibitory. Melting curve and gel electrophoresis analyses were performed to confirm that the qPCR products were of the appropriate size. No-template blank control was run for each qPCR assay and no-template blank controls gave negligible values. The copy number of 16S rRNA gene was generated using a regression equation for each assay relating the cycle threshold (Ct) value to the known number of 16S rRNA genes in the standard DNA. For each microcosm, DNA of soil was extracted with FastDNATM SPIN Kit for Soil (MP Biomedicals, Santa Ana, California, USA). And for each sample of soil DNA, qPCR reactions were run in triplicate, and the average values of triplicate qPCR reactions were used to calculate the copy number of 16S rRNA genes per gram of soil, the standard deviation of Ct values among three replicates was below 0.3.

**REFERENCES**

Amir, A., D. McDonald, J. A. Navas-Molina, E. Kopylova, J. T. Morton, Z. Z. Xu, E. P. Kightley, et al. 2017. "Deblur rapidly resolves single-nucleotide community sequence patterns." *mSystems* 2:e00191-00116.

Bokulich, N. A., S. Subramanian, J. J. Faith, D. Gevers, J. I. Gordon, R. Knight, D. A. Mills, et al. 2013. "Quality-filtering vastly improves diversity estimates from Illumina amplicon sequencing." *Naure Methods* 10:57-59.

Bolyen, E., J. R. Rideout, M. R. Dillon, N. Bokulich, C. C. Abnet, G. A. Al-Ghalith, H. Alexander, et al. 2019. "Reproducible, interactive, scalable and extensible microbiome data science using QIIME 2." *Nature Biotechnology* 37:852-857.

Callahan, B. J., P. J. McMurdie, and S. P. Holmes. 2017. "Exact sequence variants should replace operational taxonomic units in marker-gene data analysis." *The ISME Journal* 11:2639-2643.

Caporaso, J. G., C. L. Lauber, W. A. Walters, D. Berg-Lyons, C. A. Lozupone, P. J. Turnbaugh, N. Fierer, et al. 2011. "Global patterns of 16S rRNA diversity at a depth of millions of sequences per sample." *Proceedings of the National Academy of Sciences of the United States of America* 108:4516-4522.

Chen, H.Y., Jing, Q.F., Liu, X., Zhou, X.H., Fang, C.M., Li, B., Zhou, S.R. and Nie, M. 2022. "Microbial respiratory thermal adaptation is regulated by r-/K-strategy dominance." *Ecology Letters* 25**:**2489-2499.

Fierer, N., Bradford, M.A. and Jackson, R.B. 2007. "Toward an ecological classification of soil bacteria." *Ecology* 88**:**1354-1364.

Ho, A., Di Lonardo, D.P. and Bodelier, P.L.E. 2017. "Revisiting life strategy concepts in environmental microbial ecology." *FEMS Microbiology Ecology* 93**:**fix006.

Janssen, S., D. McDonald, A. Gonzalez, J. A. Navas-Molina, L. Jiang, Z. Z. Xu, K. Winker, et al. 2018. "Phylogenetic Placement of Exact Amplicon Sequences Improves Associations with Clinical Information." *mSystems* 3:e00021-00018.

Singh, B. (2018) *Soil carbon storage: modulators, mechanisms and modeling*. Academic Press, London.
